# AZD-7648, a DNA-PK Inhibitor, Induces DNA Damage, Apoptosis, and Cell Cycle Arrest in Chronic and Acute Myeloid Leukemia Cells

**DOI:** 10.3390/ijms242015331

**Published:** 2023-10-18

**Authors:** Beatriz Santos Lapa, Maria Inês Costa, Diana Figueiredo, Joana Jorge, Raquel Alves, Ana Raquel Monteiro, Beatriz Serambeque, Mafalda Laranjo, Maria Filomena Botelho, Isabel Marques Carreira, Ana Bela Sarmento-Ribeiro, Ana Cristina Gonçalves

**Affiliations:** 1 Laboratory of Oncobiology and Hematology (LOH), University Clinics of Hematology and Oncology, Faculty of Medicine (FMUC), University of Coimbra, 3000-548 Coimbra, Portugal; uc2013143376@student.uc.pt (B.S.L.); uc2018265624@student.uc.pt (M.I.C.); jjorge@fmed.uc.pt (J.J.); raquel.alves@fmed.uc.pt (R.A.); ana.raquel.monteiro@sapo.pt (A.R.M.); absarmento@fmed.uc.pt (A.B.S.-R.); 2 Coimbra Institute for Clinical and Biomedical Research (iCBR), Group of Environmental Genetics of Oncobiology (CIMAGO), Faculty of Medicine (FMUC), University of Coimbra, 3000-548 Coimbra, Portugal; beatriz.serambeque@student.uc.pt (B.S.); icarreira@fmed.uc.pt (I.M.C.); 3 Center for Innovative Biomedicine and Biotechnology (CIBB), 3004-504 Coimbra, Portugal; 4 Clinical Academic Center of Coimbra (CACC), 3000-061 Coimbra, Portugal; 5 Institute of Biophysics, Faculty of Medicine (FMUC), University of Coimbra, 3000-548 Coimbra, Portugal; 6 Cytogenetics and Genomics Laboratory, Faculty of Medicine (FMUC), University of Coimbra, 3000-548 Coimbra, Portugal; 7 Hematology Service, Centro Hospitalar e Universitário de Coimbra (CHUC), 3000-061 Coimbra, Portugal

**Keywords:** DNA damage repair, DNA-PK inhibitor, AZD-7648, myeloid leukemia, therapeutic target

## Abstract

The non-homologous end joining pathway is vital for repairing DNA double-strand breaks (DSB), with DNA-dependent protein kinase (DNA-PK) playing a critical role. Altered DNA damage response (DDR) in chronic (CML) and acute myeloid leukemia (AML) offers potential therapeutic opportunities. We studied the therapeutic potential of AZD-7648 (DNA-PK inhibitor) in CML and AML cell lines. This study used two CML (K-562 and LAMA-84) and five AML (HEL, HL-60, KG-1, NB-4, and THP-1) cell lines. DDR gene mutations were obtained from the COSMIC database. The copy number and methylation profile were evaluated using MS-MLPA and DDR genes, and telomere length using qPCR. p53 protein expression was assessed using Western Blot, chromosomal damage through cytokinesis-block micronucleus assay, and γH2AX levels and DSB repair kinetics using flow cytometry. Cell density and viability were analyzed using trypan blue assay after treatment with AZD-7648 in concentrations ranging from 10 to 200 µM. Cell death, cell cycle distribution, and cell proliferation rate were assessed using flow cytometry. The cells displayed different DNA baseline damage, DDR gene expressions, mutations, genetic/epigenetic changes, and p53 expression. Only HEL cells displayed inefficient DSB repair. The LAMA-84, HEL, and KG-1 cells were the most sensitive to AZD-7648, whereas HL-60 and K-562 showed a lower effect on density and viability. Besides the reduction in cell proliferation, AZD-7648 induced apoptosis, cell cycle arrest, and DNA damage. In conclusion, these results suggest that AZD-7648 holds promise as a potential therapy for myeloid leukemias, however, with variations in drug sensitivity among tested cell lines, thus supporting further investigation to identify the specific factors influencing sensitivity to this DNA-PK inhibitor.

## 1. Introduction

Genome integrity is of utmost importance for cell survival and homeostasis maintenance; however, in the mammalian genome, per day, it is estimated that hundreds of thousands (10^5^) of DNA lesions are generated spontaneously [1,2]. DNA double strand breaks (DSBs) are less frequent than other lesions but are difficult to repair and extremely toxic [3]. One of the major canonical mechanisms for the repair of DSBs is the non-homologous end joining (NHEJ) system [4,5]. The DNA-dependent protein kinase (DNA-PK) is a nuclear serine/threonine protein kinase formed by two components: a catalytic subunit (DNA-PKcs), encoded by the *PRKDC* gene, and a Ku heterodimer composed of Ku70 and Ku80 subunits, encoded by the *XRCC6* and *XRCC5* genes, respectively [6,7]. The most well-described function of DNA-PK is the mediation of the direct ligation of the broken ends of DNA DSBs which is primarily involved in repairing DSBs through the NHEJ repair pathway [6,7]. Besides NHEJ, this protein has also been implicated in the HR repair pathway [7]. DNA-PK has also been associated with other cellular processes, including cell cycle progression, the modulation of chromatin structure, transcriptional regulation, and telomere maintenance, and was also implicated in the cellular DNA replication stress response [4,6].

Genomic stability is crucial for faithful genome replication, therefore, cells have developed a complex DNA damage response (DDR) network to protect genome integrity, and repair damaged DNA [8]. Dysfunctional DDR alters DNA repair mechanisms and could lead to an unrepaired or misrepaired genome, thus increasing mutagenesis and genomic instability and aiding in the initiation and progression of human diseases like cancer [8,9]. Recently, DDR alterations have been implicated in the pathogenesis of various hematological malignancies, including chronic and acute myeloid leukemia (CML and AML) and therapy resistance [10,11,12]. CML is a myeloproliferative disease that is associated with the fusion gene *BCR-ABL1*, which encodes an oncoprotein with abnormally high tyrosine kinase activity [13]. AML is an aggressive and heterogeneous clonal disorder of hematopoietic stem and/or progenitor cells characterized by a block in myeloid differentiation and increased proliferation, leading to the accumulation of immature myeloid cells (myeloblasts) in bone marrow, peripheral blood, and other tissues [14,15]. Some abnormalities in DDR pathways have been shown in CML and AML, and DSB repair seems to be particularly compromised [10,16,17].

Genomic instability associated with cancer may provide targetable vulnerabilities for cancer therapy [8], and given the critical role of DNA-PK in DDR pathways, targeting this protein may be a promising strategy to increase the effectiveness of cancer therapy [6,18]. Consequently, in recent years, there has been a significant increase in the development of DNA-PK inhibitors, with several currently in clinical trials [19,20]. Even though there has been promising progress in clinical studies, there are still several obstacles to developing DNA-PK inhibitors with practical therapeutic efficacy in clinics [21,22]. One of the obstacles is the selectivity of these inhibitors [21,22]. Achieving a satisfactory selectivity for DNA-PK over strongly related kinases within the PI3K family (such as PI3Kα, β, δ, γ) and other members of the PI3K-related protein family (e.g., ataxia telangiectasia mutated (ATM) and Rad3-related (ATR)), is challenging due to the structural similarities to DNA-PK [21,22]. Addressing this concern, AZD-7648 emerges as a potent and selective DNA-PK inhibitor with an IC_50_ value of 0.6 nM in biochemical assays and a >100-fold selectivity against many closely related kinases, such as PI3Ks [4,19,21]. Currently, this drug is under clinical trials in monotherapy and combined with other anticancer compounds, like olaparib, in individuals diagnosed with advanced cancer [19,21,22]. 

Considering the doubt regarding the therapeutic potential of AZD-7648 in the treatment of myeloid leukemias, studies in this area are necessary to elucidate the relevance of this approach. Therefore, our study aimed to assess the therapeutic potential of AZD-7648 in monotherapy for myeloid leukemia treatment using in vitro models of CML and AML. We hypothesized that AZD-7648 may have promise as a new therapeutic strategy for these myeloid leukemias in monotherapy. The results from our study may help shed some light on the efficacy of this DNA-PK inhibitor and may help clarify the relevance of this therapeutic line for these diseases.

## 2. Results

### 2.1. DNA Damage Response Characterization of CML and AML Cell Lines

#### 2.1.1. Mutational Status of DDR Genes

The mutational status of genes involved in the DDR of CML and AML cell lines included in this study was retrieved from the COSMIC platform (Table 1). CML cell lines displayed a slightly lower number of mutated DDR genes (on average 11 mutated genes, ranging from 10 to 12) than AML cell lines (on average 13 mutated genes, ranging from 9 to 17). The cell line with the lowest number of mutated DDR genes was HEL, presenting 9 mutations. On the other hand, NB-4 and THP-1 were the cell lines with the highest number of mutated genes, with 17 mutated genes. 

Considering the functions associated with the mutated genes, particularly those linked with DNA repair pathways, distinct patterns are evident among the cell lines. The NER pathway was the most affected repair mechanism in the K-562, HEL, and KG-1 cell lines and the MMR pathway in THP-1 cells. LAMA-84 presented more alterations in the Fanconi anemia pathway, NB-4 in the HRR pathway, and HL-60 with the same number of mutations in the BER and HRR pathways. Further information involving the mutations can be found in the Supplementary Table S1.

#### 2.1.2. Genetic and Epigenetic Characterization of Selected DDR Genes

**Figure 1:**
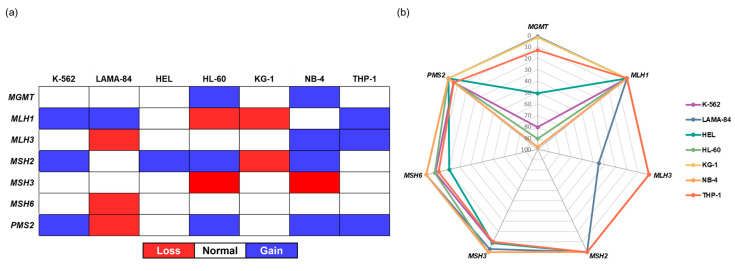
Copy number (**a**) and methylation status (**b**) of DNA damage repair genes in CML and AML cell lines. MS-MLPA results for seven DNA damage repair genes are presented as a schematic representation of the alterations in copy number and a radar chart showing the methylation status. The deletions (red) were defined as a loss of >0.20, and the amplifications (blue) as a gain of >0.20 in relation to healthy subjects. Samples with a methylation level ≥10% were classified as methylated, and two methylation ranges were determined: levels between 10% and 50% were classified as hemi-methylated, and levels between 51% and 100% were considered methylated.

The genetic and epigenetic alterations of the six genes involved in the MMR pathway, as well as one gene involved in the direct reversal of damage repair (*MGMT*), were assessed in CML and AML cell lines using methylation-specific multiplex ligation-dependent probe amplification (MS-MLPA). As shown in Figure 1a, the tested cell lines carried genetic alterations in the examined genes, and no differences were observed between the CML and AML cell lines. Among these, the *MLH1*, *MSH2*, and *PMS2* genes appeared to be the most altered, as each presented genetic abnormalities in five different cell lines. The *MSH6* gene was only altered on one CML cell line (LAMA-84). The HEL cell line presented only a genetic abnormality, specifically an amplification within the *MSH2* gene. Similarly, both K-562 (CML) and THP-1 (AML) cell lines just carried amplifications, affecting three genes each. On the other hand, the KG-1 cell line carried only deletions in the *MLH1* and *MSH2* genes. The HL-60 and NB-4 cell lines carried the highest number of genetic alterations, each with five genes affected. The *MGMT* gene exhibited the highest degree of methylation (Figure 1b). Except for the KG-1 and LAMA-84 cells, all the other cell lines were either hemi-methylated (THP-1) or methylated. The tested CML and AML cell lines were demethylated for the *MLH1*, *MSH2*, and *PMS2* genes. The *MLH3* and *MSH3* genes were only hemi-methylated in the LAMA-84 and THP-1 cell lines, respectively, and both the HEL and THP-1 cell lines were hemi-methylated in the *MSH6* gene.

#### 2.1.3. DDR-Related Genes’ Expression Levels

**Figure 2:**
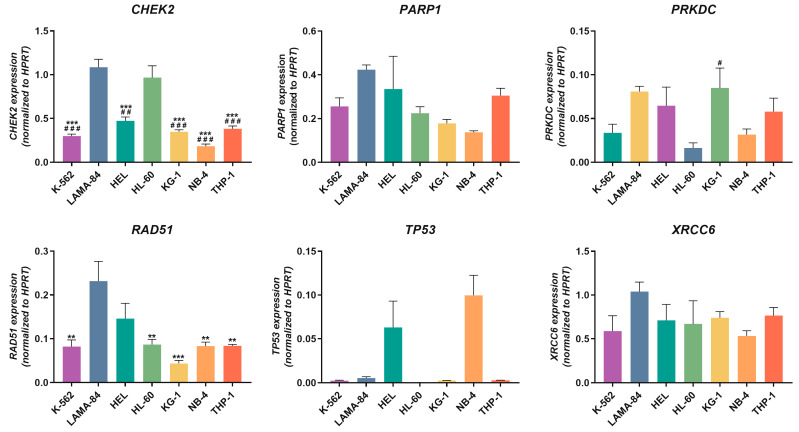
Expression levels of DDR-related genes in CML and AML cell lines. The expression of *CHEK2*, *PARP1*, *PRKDC*, *RAD51*, *TP53*, and *XRCC6* was assessed by qPCR. The results are normalized to the *HPRT* gene and represent the mean ± standard error of the mean (SEM) of 3 independent experiments. Statistical analyses were conducted using one-way ANOVA and Tukey’s multiple comparisons test or Kruskal–Wallis test and Dunn’s multiple comparisons test (*TP53*). ^#^ *p* < 0.05, ^##^ *p* < 0.01, and ^###^ *p* < 0.001 (comparison with HL-60); ** *p* < 0.01, and *** *p* < 0.001 (comparison with LAMA-84).

The baseline expression levels of the genes involved in NHEJ and alternative repair pathways (*CHEK2*, *PARP1*, *PRKDC*, *RAD51*, *TP53*, and *XRCC6*) were evaluated via qPCR. As illustrated in Figure 2, no distinct profile was observed between the CML and AML cell lines. However, the LAMA-84 cell line had the highest expression levels of *RAD51*, significantly surpassing HL-60, K-562, NB-4, and THP-1 (*p* < 0.01), and KG-1 (*p* < 0.001), which was the cell line with the lowest expression levels. Both the LAMA-84 and HL-60 cell lines presented the highest expression levels of *CHEK2*, significantly above those of HEL (*p* < 0.001 for LAMA-84; *p* < 0.01 for HL-60), K-562, KG-1, NB-4, and THP-1 (*p* < 0.001). Among these cell lines, NB-4 had the lowest expression levels. The *PRKDC* gene’s lowest expression levels were observed in HL-60 cells, significantly lower than in the KG-1 cell line (*p* < 0.05). KG-1, followed by LAMA-84 cells, exhibited the highest expression levels for this gene. Although without statistical significance, the NB-4 cell line displayed the highest *TP53* expression levels, followed by HEL. Among the remaining cell lines, except HL-60 cells that do not express the gene *TP53*, noticeably lower expression levels were observed. Lastly, regarding the *PARP1* and *XRCC6* genes, the expression levels were mostly similar across the cell lines. However, for both genes, particularly for the *PARP1* gene, LAMA-84 cells appeared to have the highest expression levels, while NB-4 had the lowest.

#### 2.1.4. p53 Protein Expression

**Figure 3:**
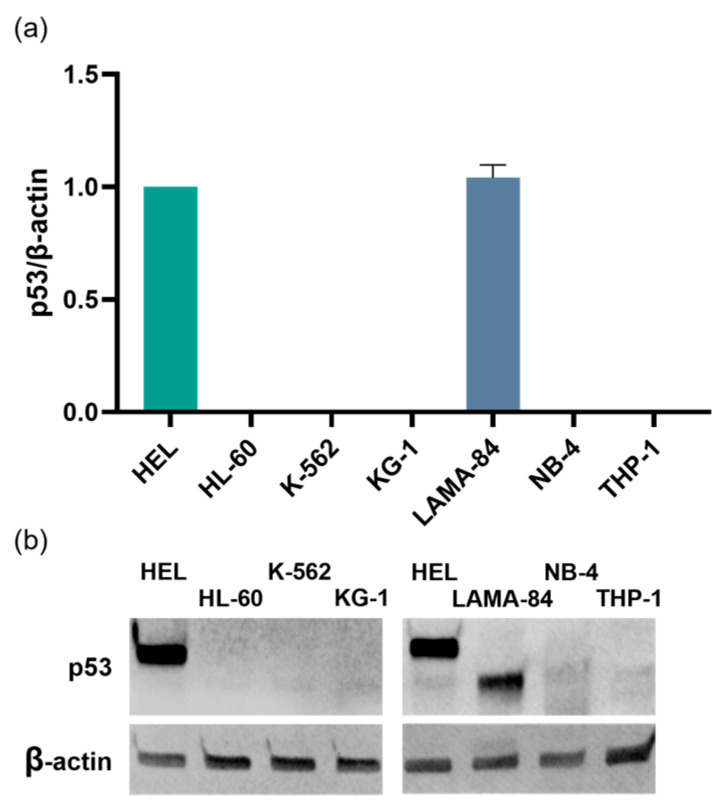
p53 protein expression in CML and AML cell lines. (**a**) The protein expression was analyzed using Western Blot and the results are presented as the ratio of the fluorescence intensities of p53 versus β-actin and the graphs represent the alteration in relation to the HEL cell line (p53/β-actin equal to 1). Results depict the mean ± SEM of 3 or 2 (NB-4 cells) independent experiments and data were statistically analyzed using the Wilcoxon signed-rank test (comparison with HEL cell line). (**b**) Representative immunoblot of p53 and β-actin for each cell line.

In addition to assessing gene expression levels, the p53 protein expression was analyzed using Western Blot. p53 expression was absent in the HL-60, K-562, KG-1, NB-4, and THP-1 cell lines, while it was exclusively detected in the HEL and LAMA-84 cells (Figure 3a). Notably, both the HEL and LAMA-84 (1.04 ± 0.06) cell lines exhibited similar levels of p53 expression. As shown in Figure 3b, in LAMA-84 cells, the p53 band exhibited a lower molecular weight compared to that in HEL cells.

#### 2.1.5. DNA and Chromosomal Damage Levels

We began by characterizing the frequency of DSB in the CML and AML cell lines, as well as in IMC (normal human lymphocyte cell line). Baseline levels of phosphorylated-H2AX (γH2AX), a biomarker of DSB, were quantified through flow cytometry (Table 2). The HEL cell line had the highest prevalence of DSBs, closely followed by NB-4. In contrast, IMC cells displayed the lowest DSB levels, which were significantly lower than the γH2AX levels observed in HEL and NB-4 (*p* < 0.001), K-562 (*p* < 0.01), and THP-1 (*p* < 0.05).

The chromosomal damage was assessed using the cytokinesis-block micronucleus cytome assay (CBMN), wherein binucleated (BN) viable cells were categorized into cells lacking chromosomal damage biomarkers (CDBs), cells with micronuclei (MNi), cells with nuclear buds (NBUDs), and cells with nucleoplasmic bridges (NPBs). Firstly, total chromosomal damage was quantified as a percentage of cells with CDBs. As detailed in Table 2, the LAMA-84 cell line demonstrated the highest CDBs score, with K-562 ranking second in terms of chromosomal damage levels. IMC cells, characterized by the lowest percentage of CDBs, exhibited significantly lower chromosomal damage compared to LAMA-84, K-562, and THP-1 (*p* < 0.001), as well as KG-1 (*p* < 0.01).

Additionally, the percentage of BN cells displaying each type of damage (MNi, NBUDs, and NPBs) was calculated (Table 2). The CML cells displayed higher chromosomal damage than the AML cell lines. The predominant type of damage was dependent on the cell line. In IMC cells, MNi were the main CDB found. Significantly higher levels of MNi were observed in IMC cells in comparison to K-562, LAMA-84, HEL, HL-60, NB-4, and THP-1 cell lines (*p* < 0.01). Within this cell line, the MNi frequency was 1.6-fold and 8.4-fold higher than the NBUDs and NPBs, respectively. The KG-1 cell line also presented MNi as the dominant chromosomal damage. Here, MNi were 1.8-fold and 2.2-fold more frequent than the NBUDs and NPBs, respectively. NPBs were the prevalent damage type in the LAMA-84 cell line, with the percentage of this damage being 2.0-fold and 1.9-fold higher than in MNi and NBUDs, respectively. When compared to IMC cells, the levels of NPBs were significantly higher, not only in the LAMA-84 (*p* < 0.001) but also in the K-562 (*p* < 0.01), HEL, KG-1, and NB-4 (*p* < 0.05) cell lines. Lastly, NBUDs were the predominant type of damage in the remaining cell lines, specifically HEL, HL-60, K-562, NB-4, and THP-1. In these cases, the percentage of NBUDs was 2.1-fold, 1.7-fold, 2.6-fold, 1.6-fold, and 1.9-fold higher than that of MNi, respectively. When comparing NPBs and NBUDs, the latter percentage was 2.4-fold, 3.5-fold, 1.9-fold, 2.3-fold, and 4.7-fold higher in the HEL, HL-60, K-562, NB-4 and THP-1 cells, respectively. Among these cell lines, THP-1 showed the highest NBUDs percentage, significantly surpassing that of the IMC cells (*p* < 0.05).

The telomere length was assessed using qPCR and normalized to the telomere length of IMC cells (Table 2). The cell lines had variability in the telomere lengths, with the KG-1 cell line having the shortest telomeres, while THP-1 shows the longest telomeres among the myeloid leukemia cell lines. All tested cell lines presented telomere lengths significantly shorter than those observed in IMC cells (*p* < 0.01).

#### 2.1.6. Double-Strand Break Repair Kinetics

**Figure 4:**
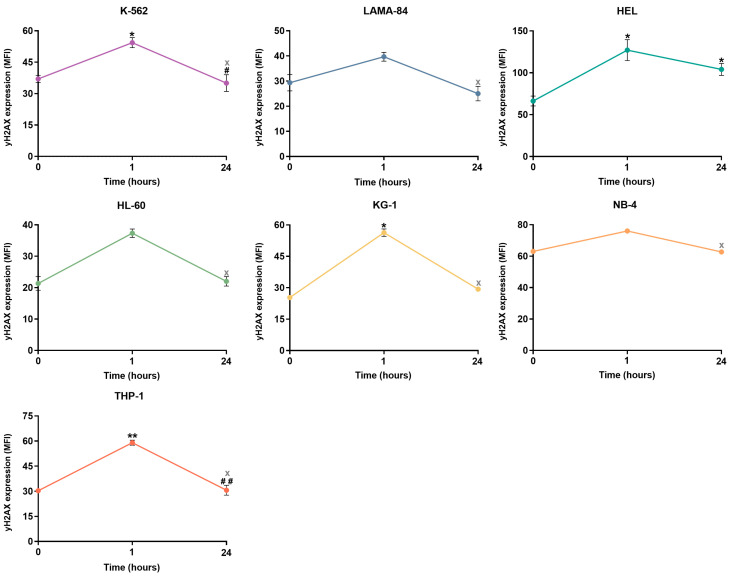
Double-strand breaks repair kinetics in CML and AML cells following H_2_O_2_ exposure. The baseline DNA damage levels of the cell lines correspond to the 0 h time point. The results represent the mean ± SEM of γH2AX mean fluorescence intensity (MFI) obtained from 3 independent experiments. Statistical analyses were performed using one-way ANOVA for repeated measures, followed by Tukey’s multiple comparisons test or Friedman test, followed by Dunn’s multiple comparisons test (KG-1, HL-60, and NB-4). * *p* < 0.05, and ** *p* < 0.01 (comparison with the 0 h time point); ^#^ *p* < 0.05, and ^##^ *p* < 0.01 (comparison with the 1 h time point). The “x” represents the absence of significant differences between 0 h and 24 h.

DSB repair kinetics were established by assessing γH2AX levels through flow cytometry at different time points following exposure to a genotoxic stimulus with H_2_O_2_. As shown in Figure 4, a significant increase in DSB damage was observed 1 h after exposure to H_2_O_2_ in the K-562 (1.5-fold, *p* < 0.05), KG-1 (2.2-fold, *p* < 0.05), and THP-1 (1.9-fold, *p* < 0.01) cells, compared to the baseline (0 hour time point). Then, this increase was followed by a significant decrease in γH2AX levels at the 24 h time point in the K-562 (1.6-fold, *p* < 0.05) and THP-1 (1.9-fold, *p* < 0.01) cell lines, reaching the baseline levels of damage. Though this decrease was not statistically significant in KG-1 cells (1.9-fold), no statistically significant differences were found between the baseline DSB levels and the levels 24 h after H_2_O_2_ exposure in any of these three cell lines. In the HL-60, LAMA-84, and NB-4 cells, while not statistically significant, a peak 1 h after the genotoxic stimuli was observed. This peak resulted from respective 1.8-fold, 1.4-fold, and 1.2-fold increases in γH2AX levels compared to the baseline. A decrease in DSB levels between the 1 and 24 h time points was observed (1.7-fold for HL-60; 1.6-fold for LAMA-84; 1.2-fold for NB-4), showing a recovery of the original γH2AX levels. Consequently, no statistical differences were observed between these cell lines’ 0 and 24 h time points. The HEL cell line showed a significant 1.9-fold increase (*p* < 0.05) in γH2AX levels 1 h after exposure to H_2_O_2_, followed by a 1.2-fold decrease at 24 h. When comparing the damage quantified at 24 h with that at the baseline levels, a significant 1.2-fold (*p* < 0.05) difference remains evident.

### 2.2. AZD-7648 Reduced Cell Density and Viability in AML Cell Lines 

**Figure 5:**
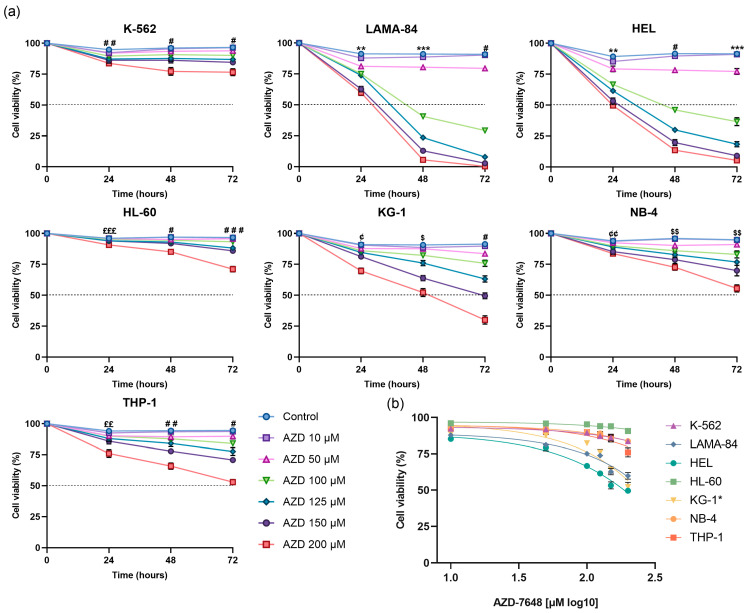
The effect of AZD-7648 (AZD) on cell viability in CML and AML cell lines. (**a**) Results are expressed as cellular viability, represented as a percentage (%) of viable cells. Results represent the mean ± SEM of 5 independent experiments. Data were statistically analyzed at each time point by comparison of the tested doses with the control using ordinary one-way ANOVA followed by Dunnett’s multiple comparisons test or Kruskal–Wallis test followed by Dunn’s multiple comparisons test. (**b**) Log dose–response viability curve of AZD-7648 after 24 or 48 (KG-1*) hours of treatment. Results represent the mean ± SEM of 5 independent experiments. ** *p* < 0.01, and *** *p* < 0.001 (control vs. 50, 100, 125, 150, and 200 μM); ^$^ *p* < 0.05, and ^$$^ *p* < 0.01 (control vs. 100, 125, 150, and 200 μM); ^#^ *p* < 0.05, ^##^ *p* < 0.01, and ^###^ *p* < 0.001 (control vs. 125, 150 and 200 μM); ^¢^ *p* < 0.05, and ^¢¢^ *p* < 0.01 (control vs. 150, and 200 μM); ^££^ *p* < 0.01, and ^£££^ *p* < 0.001 (control vs. 200 μM).

The effects of AZD-7648 on cell density and viability are represented in Supplementary Figure S1 and Figure 5, respectively. As observed in Figure 5a, AZD-7648 displayed inhibitory effects on cell viability in a dose-, time-, and cell-line-dependent manner. Despite testing a wide range of concentrations (from 10 µM to 200 µM), the inhibitor’s mathematical half-maximal inhibitory concentration (IC_50_) was not reached in the HL-60, K-562, NB-4, and THP-1 cells at any time point (IC_50_ > 200 µM). However, different behaviors were observed among these cell lines, and in HL-60 and K-562, time seemed to have limited influence on the effect of the tested doses on cell viability. In contrast, the NB-4 and THP-1 cells presented a more pronounced decrease in cell viability over time, particularly at 72 h. After 24 h of incubation, HEL cells required the lowest concentration of AZD-7648, showing an IC_50_ ranging between 150 µM and 200 µM (Figure 5b). After 48 and 72 h of incubation, the IC_50_ further decreased to 97.7 µM and 85.5 µM, correspondingly. In LAMA-84 cells, at the 24 h time point, the highest dose used (200 µM) failed to achieve a 50% decrease in cell viability. However, following 48 and 72 h of incubation required the lowest concentrations for these time points, resulting in IC_50_ values of 92.6 µM and 81.6 µM, respectively. Like this cell line, in KG-1 cells, a 50% decrease in cell viability was not reached at the tested concentrations at 24 h. At 48 h, a decrease in viability of approximately 50% was obtained with the 200 µM dose, yet the mathematical IC_50_ was only reached at 72 h, with a value of 159.9 µM.

### 2.3. Apoptosis was the Main Mechanism of Cell Death Induced by AZD-7648

**Figure 6:**
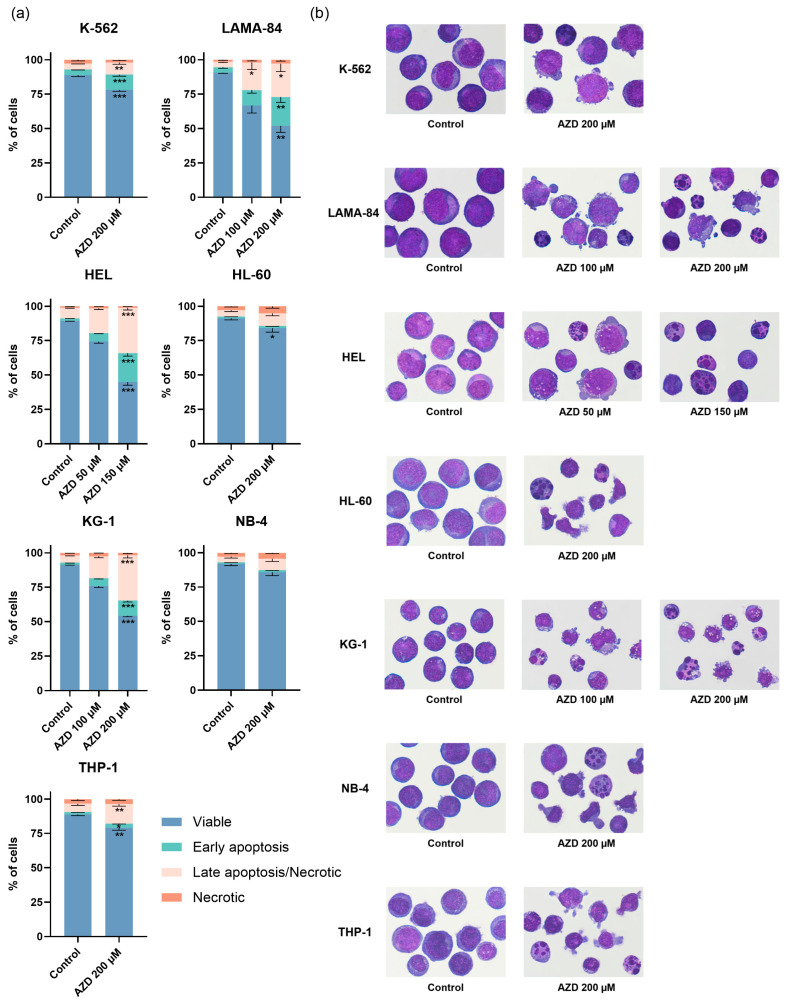
Analysis of cell death induced by AZD-7648 (AZD) in CML and AML cell lines after 24 or 48 (KG-1) hours of treatment. (**a**) The type of cell death was identified using annexin V/propidium iodide staining and analyzed using flow cytometry. Data are expressed as a percentage (%) of viable, early apoptosis, late apoptosis/necrotic, and necrotic cells and represent mean ± SEM of 5 independent experiments. Statistical analyses were performed by comparison with control, using one-way ANOVA and Dunnett’s multiple comparisons test, Kruskal–Wallis and Dunn’s multiple comparisons test, unpaired *t*-test, or Mann–Whitney test. (**b**) Cell morphology was observed by light microscopy (amplification: 1000×), and the most representative image was selected. * *p* < 0.05, ** *p* < 0.01, and *** *p* < 0.001.

We evaluated the mechanism of cell death activated by AZD-7648 at the doses that induced a decrease in cell viability of approximately 25% and 50% within the doubling time of each cell line (24 h for all except KG-1, which had a doubling time of 48 h) (Figure 6). No statistical differences were observed in the HEL or KG-1 cells following treatment with 50 µM and 100 µM of AZD-7648, respectively. Still, compared to the control, there was a corresponding increase in the percentage of cells in early apoptosis, by 3.4-fold and 4-fold, and in late apoptosis/necrosis by 2.4-fold and 3.1-fold. After treatment with the higher doses, a significant rise in early apoptotic (HEL: 11.7-fold, *p* < 0.001; KG-1: 7.9-fold, *p* < 0.001) and late apoptotic/necrotic cells (HEL: 4.3-fold, *p* < 0.001; KG-1: 6.3-fold, *p* < 0.001) was observed in both cell lines. This increase was linked to a decrease in the percentage of viable cells (*p* < 0.001). In the LAMA-84 cell line, treatment with 100 µM of AZD-7648 led to an increase in the percentage of early apoptotic (2.8-fold) and late apoptotic/necrotic (5-fold, *p* < 0.05) cells, compared to untreated cells. The treatment with 200 µM of the inhibitor significantly increased the early and late apoptotic LAMA-84 cells (5.3-fold, *p* < 0.01; 6.1-fold, *p* < 0.05, respectively). The effect of the highest tested dose was evaluated in the cell lines where the IC_50_ was not reached (HL-60, K-562, NB-4, and THP-1). Mainly, the K-562 and THP-1 cells treated with 200 µM of the inhibitor showed a decrease in viable cells, accompanied by a significant increase of 2.6-fold (*p* < 0.001) and 1.7-fold (*p* < 0.05) in the early apoptotic and 2.1-fold (*p* < 0.01) and 2.4-fold (*p* < 0.01) in the late apoptotic/necrotic cells, respectively, compared to the control. Despite the absence of statistical difference, a 2-fold increase in the late apoptotic/necrotic population was noticeable in the HL-60 and NB-4 cells following inhibitor treatment (Figure 6a). 

In agreement with these results, cells treated with AZD 7648 showed morphological aspects typical of apoptosis, like blebbing, nuclear fragmentation, and cellular contraction (Figure 6b). After treatment with the inhibitor, it was also possible to observe an increase in vacuolization and cell shape and cytoplasm changes.

**Figure 7:**
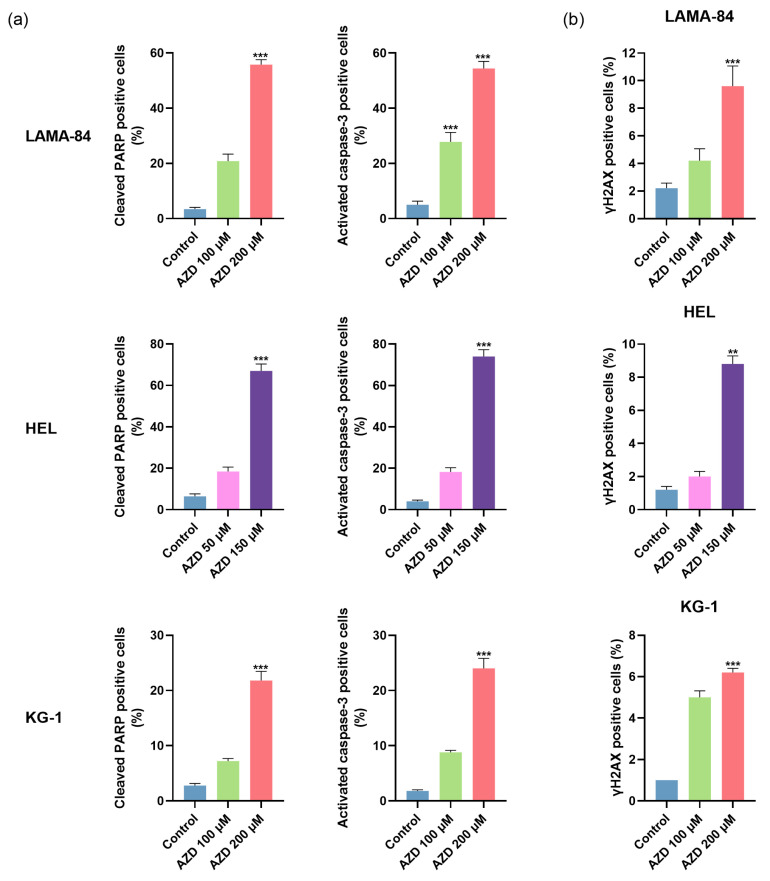
The expression levels of cleaved PARP and activated caspase-3 (**a**) and phosphorylated-H2AX (γH2AX) (**b**) in LAMA-84, HEL, and KG-1 cell lines after treatment with AZD-7648 (AZD). Results were obtained after 24 or 48 (KG-1) hours of incubation and represent mean ± SEM of 5 independent experiments. Data were statistically analyzed by comparison with control using Kruskal–Wallis followed by Dunn’s multiple comparisons test or one-way ANOVA followed by Dunnett’s multiple comparisons test. ** *p* < 0.01, and *** *p* < 0.001.

Afterwards, we quantified the levels of cleaved PARP and activated caspase-3 in the cell lines where the IC_50_ was reached (LAMA-84, HEL, and KG-1), further supporting apoptosis as the main cell death mechanism due to a significant increase in cleaved PARP and activated caspase-3-positive cells (Figure 7a). Following 24 h of incubation, an increase in HEL positive cells for cleaved PARP of 2.9-fold and 10.5-fold (*p* < 0.001) was observed after treatment with 50 µM and 150 µM of AZD-7648, respectively, compared to untreated cells. Similarly, in the LAMA-84 cell line, the inhibitor increased the percentage of cells with cleaved PARP (100 µM: 6.1-fold; 200 µM: 16.4-fold, *p* < 0.001). In the KG-1 cell line, after 48 h of treatment, similar results were observed, with a 2.6-fold, and 7.8-fold (*p* < 0.001) increase in the cleaved PARP levels after treatment with 100 µM and 200 µM, respectively. Likewise, an increase in activated caspase-3-positive cells was observed after 24 h of treatment (HEL: 4.6-fold for 50 µM and 18.5-fold, *p* < 0.001 for 150 µM; LAMA-84: 5.6-fold, *p* < 0.001 for 100 µM and 10.9-fold, *p* < 0.001 for 200 µM) compared to the control. The KG-1 cell line presented a similar trend after 48 h of treatment with 100 µM and 200 µM of AZD-7648, increasing the percentage of activated caspase-3 cells by 4.9-fold and 13.3-fold (*p* < 0.001), correspondingly. 

As represented in Figure 7b, the treatment with AZD-7648 also led to an increased percentage of cells positive for γH2AX (a marker for DNA damage) compared to untreated cells. This increase was particularly evident in the HEL cell line after treatment with 150 µM (7.3-fold, *p* < 0.01) for 24 h. The tested doses in LAMA-84, 100 µM, and 200 µM increased γH2AX-positive cells by 1.9-fold and 4.4-fold (*p* < 0.001), respectively. For the KG-1 cell line, treatment with 100 µM and 200 µM of AZD-7648 for 48 h led to similar increases in γH2AX-positive cells (5-fold and 6.2-fold, *p* < 0.001, respectively).

### 2.4. AZD-7648 Promotes Cell Cycle Arrest in G_0_/G_1_ Phase and Reduces Cell Proliferation

Besides the cytotoxic effect, the cytostatic impact of AZD-7648 was also assessed after 24 or 48 (KG-1) hours of treatment with AZD-7648 (Table 3). This DNA-PK inhibitor arrested cells within the G_0_/G_1_ phase, leading to an increased percentage of cells in this cell cycle phase compared to untreated cells. The treatment with the IC_25_ dose induced a significant increase in the proportion of cells in the G_0_/G_1_ phase in HEL (1.7-fold, *p* < 0.05), KG-1 (2-fold, *p* < 0.001), and LAMA-84 (1.3-fold, *p* < 0.001) cells. A similar trend in cell cycle distribution was observed for these cell lines after exposure to higher doses (HEL: 1.8-fold, *p* < 0.05 for 150 µM; KG-1: 1.8-fold for 200 µM; LAMA-84: 1.3-fold, *p* < 0.001 for 200 µM). The HL-60, NB-4 and THP-1 cell lines exhibited a significant increase of 2.2-fold (*p* < 0.001), 1.9-fold (*p* < 0.001), and 1.3-fold (*p* < 0.001), correspondingly, in G_0_/G_1_ cells after the treatment with 200 µM of AZD-7648. On the contrary, in K-562 cells, the cell cycle distribution remained similar between the control and treated cells. Additionally, the presence of an apoptotic peak (Sub-G_1_), corresponding to the DNA fragmentation typical of apoptotic cells, was observed across all tested concentrations of AZD-7648, except in HL-60, particularly in the HEL cells treated with 150 µM (*p* < 0.01) and KG-1 cells treated with 200 µM (*p* < 0.01), thus reinforcing the induction of apoptosis as demonstrated in previous studies (Figure 6 and Figure 7).

**Figure 8:**
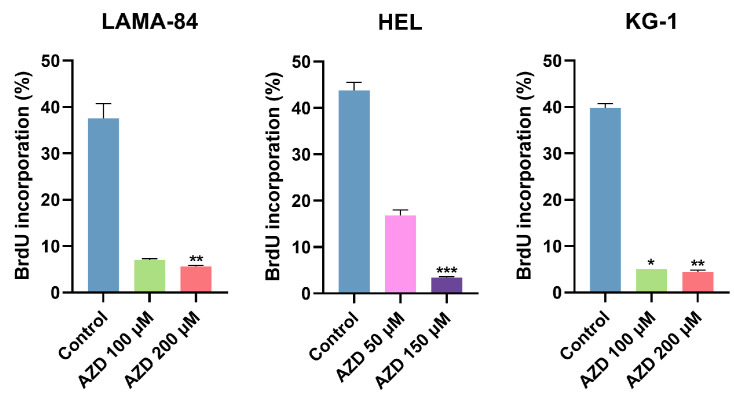
Effect of AZD-7648 (AZD) on cell proliferation of LAMA-84, HEL, and KG-1 cell lines. Results were obtained after 24 or 48 (KG-1) hours of incubation and represent the percentage (%) of BrdU incorporation. Data are expressed as mean ± SEM of 5 independent experiments and were statistically analyzed by comparison with control, using Kruskal–Wallis followed by Dunn’s multiple comparisons test. * *p* < 0.05, ** *p* < 0.01, and *** *p* < 0.001.

The incorporation of BrdU, a marker for cell proliferation, was assessed in the LAMA-84, HEL, and KG-1 cell lines after treatment with different concentrations of AZD-7648 for 24 h, or 48 h in the case of KG-1 (Figure 8). Compared to the control, the AZD-7648-treated cells showed a decrease in cell proliferation. In HEL cells treated with 50 µM of AZD-7648 and LAMA-84 with 100 µM, the percentage of BrdU incorporation decreased 2.6-fold and 5.4-fold, respectively. This reduction was more pronounced in these cell lines when treated with a higher concentration (HEL: 12.9-fold, *p* < 0.001; LAMA-84: 6.7-fold, *p* < 0.01). In the KG-1 cell line, treatment with 100 µM and 200 µM of the inhibitor for 48 h resulted in significant decreases of 8-fold (*p* < 0.05) and 9-fold (*p* < 0.01) in cell proliferation, respectively.

## 3. Discussion

Significant progress has been made in the CML and AML treatment options. However, CML patients develop resistance or are intolerant to tyrosine kinase inhibitors [13] and AML current therapies are insufficient to induce the complete remission of the disease [23]. Thus, developing new and better strategies to treat these diseases is still needed. Altered DDR and increased DNA damage are critical features of genetic instability that are presumably implicated in the pathogenesis and progression of CML and AML, and this genetic instability may be a target for therapy. Moreover, an excessively active NHEJ system was proposed as a potential mechanism for chromosomal instability in myeloid leukemias [17]. Therefore, in this study, we assessed the therapeutic potential of AZD-7648, a DNA-PK inhibitor, in CML and AML in vitro models. 

Of the tested cell lines, the cellular density and viability decrease induced by AZD-7648 was more evident in the LAMA-84, HEL, and KG-1 cell lines. A decrease in cell viability after treatment with a DNA-PK inhibitor was also observed in previous studies [4,8,24]. As previously stated, DNA-PK is predominantly involved in the NHEJ repair pathway by repairing DNA DSBs [6,7]. After the appearance of a DNA DSB, one of the first events is the phosphorylation of histone H2AX (γH2AX) on serine 139 by ATM, ATR, and DNA-PK kinases [25]. Since this phosphorylation is specific to DSBs, the quantitative assessment of this damage can be carried out by detecting γH2AX levels [26]. A previous study showed that tumor cells that retain γH2AX levels higher than the baseline 24 h after treatment with DNA damaging agents are not likely to survive [27]. Hence, although not verified, the authors hypothesize that neoplastic cells that decrease γH2AX levels to baseline upon genotoxic exposure could be drug-resistant [27]. Efficient DNA damage repair in cancer cells has been viewed as a crucial mechanism of therapeutic resistance and a contributor to chemotherapy failure [28,29]. In our study, the HEL cells presented the highest γH2AX levels and the lowest repair capacity of all tested cell lines, which could indicate compromised DSB repair. This impairment should make this cell line more sensitive to DSB-repair-targeted drugs, such as AZD-7648, as observed in this work.

The NB-4 cell line also presented high γH2AX levels. This cell line has the highest number of mutated genes related to HR repair (*BRCA1*, *RAD51D*, *PAXIP1*, and *RBBP8*), three of which are only mutated in this cell line. Considering that the HR repair pathway is responsible for the accurate repair of DSBs [30], the mutations may constitute a possible explanation for the baseline accumulation of DSBs on the cell line. The LAMA-84 and K-562 cell lines exhibited the highest levels of chromosomal damage. These cell lines are models of CML in blast crisis and present the fusion gene *BCR-ABL1*. This fusion gene has been linked to resistance to apoptosis and the down-regulation of DNA-PKcs, leading to deficient DNA repair [31]. Furthermore, blastic transformation in CML has been associated with DDR alterations, the generation and accumulation of DSBs, and error-prone DSB repair [12]. These alterations contribute to chromosomal aberration formation and subsequent accumulation [32], which could explain the greater levels of chromosomal damage found in these cell lines. The three types of scored chromosomal damage have different origins. MNi are formed as a result of clastogenic changes, such as chromosome breakage, or/and aneugenic changes, such as whole chromosome loss [33,34]. NBUDs are formed due to the elimination of amplified DNA and/or DNA repair complexes, and NPBs are formed because of DNA misrepair and/or telomere end-fusions [35]. Hence, the percentage of each chromosomal damage can show the most frequent damage and, consequently, its origin. The more prevalent chromosomal damage was dependent on the cell line. NPB formation was correlated with telomere length, and this biomarker was proposed to be used as a surrogate measure of critically short telomeres [35]. A previous article showed that shorter telomeres are associated with higher levels of NPBs [36]. Consequently, it would be expected that LAMA-84, the cell line with the highest proportion of NPBs, would possess the shortest telomeres. However, we observed that the KG-1 cell line had the shortest telomeres, yet MNi was the prevalent type of damage in this cell line. Interestingly, in accordance with the article, the IMC cell line presented the longest telomeres and the lowest percentage of NPBs.

A previous study by Jdey et al. showed that sensitivity to AsiDNA, a DDR inhibitor that acts on enzymes involved in the HR, NHEJ, BER, and single strand breaks (SSB) repair pathways, was associated with high basal levels of MNi [37]. Among the myeloid leukemia cell lines, KG-1 presented the highest frequency of MNi and was also one of the three cell lines displaying sensitivity to AZD-7648. In addition to HEL and KG-1, the LAMA-84 cell line also presented heightened sensitivity to AZD-7648 treatment. Strikingly, these latter two cell lines presented the highest expression levels of the *PRKDC* gene, responsible for encoding DNA-PK [6,7], as well as comparatively lower γH2AX baseline levels, potentially indicative of a more active NHEJ repair pathway. This enhanced NHEJ activity might explain their heightened sensitivity to DNA-PK inhibitors, such as AZD-7648, as observed in our study. Additionally, previous studies proposed that NHEJ is upregulated in cells deficient in the Fanconi anemia pathway and that, after replicative stress, DNA-PK inhibitors sensitize Fanconi anemia-deficient cells [38,39]. The LAMA-84, HEL, and KG-1 cells presented mutations in the Fanconi anemia genes, which may imply, in addition to the previously mentioned factors, a reliance on the NHEJ repair pathway.

The HL-60 and K-562 cell lines, closely followed by the NB-4 and THP-1 cells, demonstrated resistance to AZD-7648. As is known, the same type of DNA damage can be repaired by different repair pathways, thus showing the redundancy in DNA repair [40,41,42]. This redundancy potentially accounts for the inhibitor resistance in these cell lines, as they could rely on repair pathways that, contrary to NHEJ, are not as dependent on DNA-PK activity, such as HR, alternative-NHEJ, or single-strand annealing. Furthermore, the DNA repair mechanisms also exhibit a certain level of interdependence [40]. Interestingly, HL-60, K-562, NB-4, and THP-1 collectively exhibited an amplified copy number of MMR pathway genes across a broader spectrum of genes. This result was coupled with clearly low methylation levels on these genes across all cell lines—except for *MLH3* in the LAMA-84 cell line. Elevated methylation levels were mainly detected in the direct reversal damage repair gene (*MGMT*). The copy number amplification potentially triggers the increased expression of corresponding MMR proteins. This increase could result in a more effective MMR pathway, increasing the cell’s capacity to repair DNA mismatches and errors caused by DDR inhibitors. Furthermore, MMR and HR pathways are connected, and studies showed that MMR proteins, such as MSH2 and MSH3, are involved in HR repair choice [43,44]. This link could suggest an enhanced HR activity within the resistant cell lines. This trait might allow them to effectively repair damaged DNA, even in the presence of a DNA-PK inhibitor, resulting in AZD-7648 resistance. The resistance to AZD-7648 could also be explained by reduced DNA-PK activity. 

A previous study proved that a population of p53-deficient cells was not sensitive to DNA-PK inhibition [45]. This work showed that, in p53-deficient cells, the overexpression of DNA Polymerase Theta (Pol θ) occurs, mediating an alternative end-joining repair pathway that is even hyperactivated by DNA-PK inhibition [45]. This finding could explain the resistance observed in K-562, HL-60, NB-4, and THP-1 cells, as none of these cell lines expressed p53. A combination of DNA-PK and Pol θ inhibitors seems to be a viable approach to re-sensitize these cell lines [45,46]. Although the HEL and LAMA-84 cells displayed both p53 expression and sensitivity to AZD-7648, KG-1 exhibited sensitivity to the inhibitor despite the absence of p53 expression. In this cell line, the sensitivity to the inhibitor could be dependent on alternative DNA repair mechanisms or genetic factors unrelated to p53, showcasing the complex interplay of multiple pathways influencing drug response.

Genetic alterations in the *TP53* gene were detected in all cell lines; however, the nature and severity of these mutations varied depending on the specific cell line. In the case of the HL-60 cell line, the extensive deletion of the *TP53* gene was detected [47] and corroborated by the absence of *TP53* expression. In the KG-1 cell line, the *TP53* mutation originated from a substitution in a non-coding region that functions as a splice donor site. In the HEL and NB-4 cells, a missense mutation led to the replacement of one amino acid, and the SIFT (Sorting Intolerant From Tolerant) score predicted that the mutations are likely to have a harmful or damaging effect on the protein’s function. For the K-562 and THP-1 cell lines, the *TP53* mutation resulted in a frameshift due to an insertion or deletion, respectively. Notably, these mutations in the above-mentioned five cell lines are located within the p53 DNA binding domain. Lastly, in LAMA-84 cells, a nonsense mutation is found in the p53 tetramerization domain, which could account for the observed lower molecular weight of p53 in this cell line. All these mutations are considered oncogenic (K-562, NB-4, and THP-1 cells) or likely oncogenic (LAMA-84, HEL, and KG-1 cells).

As previously shown by Fok et al., the treatment with AZD-7648 induces apoptosis [4]. In line with these conclusions, our results presented a decrease in cellular viability attributed to increased apoptosis. This effect was proved by a significant increase in the proportion of apoptotic cells and an increase in cells displaying cleaved PARP and activated caspase-3—known biomarkers of apoptosis. A previous study using a different DNA-PK inhibitor showed a slight increase in induced caspase-3 or PARP cleavage in the cells after treatment [24]. We also observed that treatment with AZD-7648 increases the percentage of cells with H2AX phosphorylated. Since the phosphorylation of this histone is a biomarker of DSBs, it was possible to conclude that AZD-7648 induced DNA DSBs. Since AZD-7648 inhibits a protein involved in NHEJ [6,7] and, consequently, DSB repair, the increase in phosphorylated H2AX was expected. 

Besides the cytotoxic effect observed after treatment with AZD-7648, this inhibitor also presented a cytostatic effect. This effect was observed as an increase in the percentage of cells in the G_0_/G_1_ phase. Cell cycle arrest in this phase allows cells to repair damaged DNA or follow the apoptotic pathway [48]. In addition, the decrease in BrdU incorporation corroborated this result observed after the treatment with AZD-7648. Cell cycle arrest in G_0_/G_1_ and reduction in BrdU incorporation after treatment with a DNA-PK inhibitor were also previously observed by Hafsi et al. [24]. The cytostatic effect was expected, since it was observed that lower doses (10 μM and 50 μM), as well as higher doses in K-562 and HL-60, led to a decrease in cellular density (Supplementary Materials) while having little to no effect on cellular viability. Nevertheless, in the K-562 cell line, treatment with the DNA-PK inhibitor affected cell density without triggering significant alterations in the distribution of cells across the cell cycle phases. This phenomenon could suggest that DNA-PK inhibition might provoke responses beyond the cell cycle regulation in this cell line. In response, these cells could trigger compensatory mechanisms—such as autophagy and metabolic adaptations—to sustain cell cycle progression even in the presence of DNA damage caused by the inhibitor. These mechanisms, in turn, might contribute to changes in cell survival and density without necessarily affecting the cell cycle distribution. This response could also contribute to the observed resistance within this cell line, as cells might prioritize survival over proliferation. The induction of autophagy after treatment with DNA-PK inhibitors was, indeed, observed in previous studies [49,50]. In addition, DNA-PK has roles beyond the cell cycle, such as in promoting transcription and influencing gene expression [4,6]. Therefore, DNA-PK inhibition could impact these functions, which might affect cell survival and density downstream. 

While previous works explored the potential of DNA-PK inhibition as a therapeutic pathway in AML, limited effects were observed when treating the cell lines only with this inhibitor [51,52,53]. In these studies, the DNA-PK inhibitors M3814 (nedisertib, peposertib) [51,52], NU7441, or CC-115 [53] were used. M3814 and NU7441 are potent and selective DNA-PK inhibitors, with IC_50_ values of less than 3 nM and 14 nM, respectively [22,54]. CC-115, on the other hand, functions as a dual inhibitor targeting both mTOR and DNA-PK, with IC_50_ values of 21 nM and 13 nM, respectively [55]. In the context of this study, the DNA-PK inhibitor AZD-7648 was used. With a robust inhibitory potential (IC_50_ of 0.6 nM), AZD-7648 also presents a high selectivity against closely related kinases (e.g., ATM, ATR, PI3Kα, PI3Kβ, PI3Kδ) [4,19,21]. This distinctive selectivity might elucidate the observed impact on cell density and viability after administering this inhibitor in monotherapy. Hence, the use of AZD-7648 could account for the divergent results compared to the use of other DNA-PK inhibitors.

Although this study gives information about the therapeutic potential of AZD-7648 in myeloid leukemias, it is essential to identify some limitations that could provide direction for future research. Firstly, this study used in vitro cell culture models, which do not fully represent the complexity of leukemia in a living organism and the diversity of genetic and molecular alterations found in patients. Therefore, future research could use primary cell line cultures and in vivo studies to better assess the drug’s effectiveness and safety. Furthermore, in this study, a human lymphocyte cell line was used as the model for normal cells, instead of a myeloid cell line. This choice was due to the difficulties associated with establishing and maintaining normal myeloid cell lines, making the lymphocyte cell line a better alternative. Secondly, at the tested concentrations, AZD-7648, in addition to inhibiting DNA-PK, may also present off-target effects on other kinases involved in DSB repair, such as ATM and ATR. Hence, in future studies, it would be valuable to investigate the status and activity of these proteins to clarify the mechanism of action of this drug at the tested concentrations. Lastly, the differences in drug sensitivity observed among the tested cell lines highlight the heterogeneity of leukemia cells and the specific factors influencing these differences should be studied.

## 4. Materials and Methods

### 4.1. Cell Culture 

We used two CML in blast crisis cell lines (K-562 and LAMA-84) and five AML cell lines: HEL (human erythroleukemia), HL-60 (acute myeloid leukemia), KG-1 (human erythroleukemia), NB-4 (acute promyelocytic leukemia), and THP-1 (acute monocytic leukemia). K-562, HEL, HL-60, and KG-1 were purchased from the ATCC, and LAMA-84, NB-4, and THP-1 were purchased from the German Collection of Microorganisms and Cell Cultures (DSMZ). Additionally, an immortalized normal human lymphocyte cell line (IMC) was used and obtained from the Cytogenetics and Genomics Laboratory, Faculty of Medicine, University of Coimbra. All the cells were cultured in Roswell Park Memorial Institute 1640 medium (RPMI-1640; Gibco, Invitrogen, Waltham, MA, USA) supplemented with 10% or 20% (IMC) of heat-inactivated fetal bovine serum (FBS; Gibco, Invitrogen), 2 mM of L-glutamine, 100 U/mL of penicillin, and 100 μg/mL of streptomycin (Gibco, Invitrogen). Cells were grown at initial densities of 0.3 (HL-60), 0.4 (HEL and NB-4), or 0.5 (IMC, K-562, KG-1, LAMA-84, and THP-1) × 10^6^ cells/mL and maintained at 37 °C in a humidified atmosphere containing 5% CO_2_. The cell lines were negative for Mycoplasma and were authenticated using short tandem repeat analysis.

AZD-7648 (AZD—DNA-PK inhibitor) was purchased from MedChemExpress and was prepared in dimethyl sulfoxide (DMSO). Cell lines were incubated in the absence or presence of increasing doses of AZD-7648, ranging from 10 to 200 µM, for different periods depending on the assay. The stocks were prepared in the medium on the day of use to avoid DMSO cytotoxicity and ensure consistency between conditions.

### 4.2. DDR Pathways Mutations Database Analysis

The DDR pathways mutations data were retrieved from the COSMIC platform, available at https://cancer.sanger.ac.uk/cell_lines accessed on 10 August 2023. The genes were grouped according to human DNA repair genes (mdanderson.org accessed on 10 August 2023). The oncogenic classification and the mutations coding impact were obtained from two databases, the Franklin by Genoox (https://franklin.genoox.com/clinical-db/home accessed on 10 August 2023) and the Varsome (https://varsome.com/ accessed on 10 August 2023). Lastly, the SIFT and PolyPhen scores and predictions were gathered from the Variant Effect Predictor (https://useast.ensembl.org/Homo_sapiens/Tools/VEP accessed on 10 August 2023). 

### 4.3. Methylation-Specific Multiplex Ligation-Dependent Probe Amplification (MS-MLPA)

MS-MLPA (ME011) probe panel was applied as described in the manufacturer’s protocol (MRC-Holland, Amsterdam, Holland). This panel determines the methylation status of selected GCGC sites in the promoter regions of human repair genes. It also detects deletions or duplications in the human 3′ region of these genes. Succinctly, 100 ng of each DNA sample was used, and the amplification products were detected and measured using capillary electrophoresis on an ABI-3500 genetic analyzer (Applied Biosystems, Foster City, CA, USA). Subsequently, the results were evaluated using Coffalyser software version v.220513.1739 (MRC-Holland, Amsterdam, The Netherlands). Each MS-MLPA assay also included three control DNA samples obtained from healthy individuals and negative control (no template control). Methylation analysis involved the identification of distinct methylation ranges: demethylation (0–9%), hemi-methylation (10–50%), and methylation (51–100%). For copy number analysis, a value below 0.8 was categorized as deletion (copy number loss), while a value surpassing 1.2 was categorized as duplication (copy number gain).

### 4.4. Gene Expression Analysis

The total RNA of the seven cell lines was extracted using TripleXtractor (GRiSP, Porto, Portugal) and converted to cDNA using Xpert cDNA Synthesis Kit (GRiSP), according to the manufacturer’s protocol. Expression of six DDR genes (*CHEK2*, *PARP1*, *PRKDC*, *RAD51*, *TP53*, and *XRCC6*) was evaluated by qPCR (Xpert Fast SYBR, GRiSP) in a QuantStudio™ 3 System (ThermoFisher Scientific, Waltham, MA, USA). Hypoxanthine phosphoribosyltransferase (*HPRT*) was used as the endogenous control gene. Relative gene expression was calculated using the 2^−ΔΔCt^ formula, and the results represent the fold change mean ± SEM of three independent experiments. 

### 4.5. p53 Expression

The expression of p53 was evaluated using Western Blot, following established protocols [56]. Briefly, total protein extracts from the CML and AML cell lines were prepared and stored at −80 °C. Protein quantification was carried out using the Pierce BCA Protein Assay Kit (ThermoFisher Scientific, Waltham, MA, USA). Next, sodium dodecyl-sulfate polyacrylamide gel electrophoresis (SDS-PAGE) was employed to separate the proteins, which were then transferred onto polyvinylidene difluoride membranes (Bio-Rad, Basel, Switzerland) with a Transfer-Blot Turbo Transfer System (Bio-Rad). Incubation with primary antibodies, namely anti-β-actin (Sigma-Aldrich, St. Louis, MO, USA, A5316) and anti-p53 DO7 (Santa Cruz Biotechnology, Inc., Dallas, TX, USA, sc-47698), was performed overnight at 4 °C with continuous agitation. Incubation with the secondary antibody (anti-mouse, GE Healthcare, Chicago, IL, USA, RPN5781) was performed for 90 min at room temperature. Lastly, the blots were stained with fluorescent reagent elemental chlorine-free (ECF Western Blotting Reagent Pack, Amersham Biosciences, Buckinghamshire, UK) and developed using the ChemiDoc™ Imaging Systems (Bio-Rad). The fluorescence quantification was performed using the ImageJ Software v.1.50i. The results represent the mean ± SEM of the ratio of the fluorescence intensities of p53 versus β-actin from three independent experiments.

### 4.6. γH2AX Expression Assessment

The γH2AX expression levels were evaluated through flow cytometry. Briefly, 0.5 × 10^6^ cells were washed, fixed, and permeabilized (Fixation and Permeabilization Kit; ImmunoStep, Salamanca, Spain). Afterwards, cells were stained with 1 µL of anti-γH2AX antibody (BD Pharmingen, Becton Dickinson, Franklin Lakes, NJ, USA) for 20 min, in the dark, at room temperature. Lastly, cells were washed, resuspended in PBS, and analyzed in a FACS Calibur flow cytometer (Becton Dickinson). Through CellQuest software v.3.3 (Becton Dickinson), 50,000 cells were acquired, and data were analyzed using Paint-a-Gate software v.3.0 (Becton Dickinson). The results were expressed in mean fluorescence intensity (MFI, arbitrary units) and represented mean ± SEM of three independent experiments.

### 4.7. Cytokinesis-Block Micronucleus Cytome Assay (CBMN)

Chromosomal damage biomarkers were evaluated using the cytokinesis-block micronucleus cytome assay. Cell lines were cultured at their optimal density and incubated for 24 h with 4.5 µg/mL or 3 µg/mL (NB-4) of cytochalasin-B (Sigma-Aldrich, St. Louis, MO, USA). Cells were then treated and analyzed as described by Costa et al. [57]. Chromosomal damage was evaluated by scoring 500 viable binucleated cells for the presence of MNi, NBUDs, and NPBs. These endpoints were scored following the morphological criteria recommended by Fenech [35]. The results show the mean ± SEM obtained from five independent experiments for the proportion of cells with chromosomal damage and the percentages of each biomarker.

### 4.8. Telomere Length Quantification

The genomic DNA used was isolated using the salting out method. According to the manufacturer’s instructions, the telomere length of the tested cell lines was assessed using Relative Human Telomere Length Quantification qPCR Assay Kit (ScienCell, Carlsbad, CA, USA). Briefly, two primer sets were used: the telomere primer that recognizes and amplifies telomere sequences and the single copy reference primer that serves as a reference for data normalization. Equal amounts of DNA (5 ng/µL) were used for each reaction. qPCR reactions were performed in a QuantStudio™ 3 System (ThermoFisher Scientific, Waltham, MA, USA). The telomere length of the IMC cell line was used as a reference for calculating telomere length. Relative telomere length was calculated using the 2^−ΔΔCt^ formula. The results represent the fold change mean ± SEM of three independent experiments.

### 4.9. Double-Strand Breaks Repair Evaluation

The cell lines were cultured at their optimal density and were exposed, for 30 min, to H_2_O_2_ (Sigma-Aldrich, St. Louis, MO, USA) (K-562, HL-60, KG-1, THP-1 = 17.5 μM; NB-4 = 13.5 μM; HEL = 10 μM; LAMA-84 = 7.5 μM) in a humidified atmosphere containing 5% CO_2_. The repair capacity of double-strand breaks was evaluated by assessing its kinetics through the expression levels of γH2AX. Following the genotoxic exposure, the cell suspensions were harvested, centrifuged for 5 min at 42× *g*, and cultured in a new medium. Then, at 1 h and 24 h post-exposure to H_2_O_2_, 0.5 × 10^6^ cells were collected and subjected to a 5 min PBS wash at 300× *g*. The levels of γH2AX were quantified using the intracellular staining protocol outlined in Section 4.6. The results were presented as MFI measurement’s mean ± SEM of three independent experiments.

### 4.10. Cell Density and Viability Analysis

The trypan blue exclusion assay assessed cell density and viability for 72 h. Briefly, after every 24 h of incubation, an aliquot of cells was harvested and stained with an equal volume of trypan blue (Sigma-Aldrich), loaded in a hemocytometer, and counted resorting to the Countess™ II Automated Cell Counter (ThermoFisher Scientific). The instrument automatically calculates cell density and viability based on the number of viable cells per mL or the percentage of viable cells, respectively. The results were expressed as mean ± SEM of five independent experiments.

### 4.11. Cell Death Analysis

Cell death was assessed using flow cytometry, through annexin V (AV) and propidium iodide (PI) double staining, and by morphological analysis using optic microscopy. Succinctly, after 24 h or 48 h (KG-1) of incubation in the absence or presence of 50 µM, 100 µM, 150 µM, or 200 µM of AZD-7648, 0.5 × 10^6^ cells were collected and washed with PBS by centrifugation at 1000× *g* for 5 min. Afterwards, cells were resuspended in 100 μL of AV binding buffer and incubated with 5 μL of AV-APC (Biolegend, San Diego, CA, USA) and 1 μL of PI (Biolegend) for 15 min in the dark at room temperature. Cells were then diluted in 300 µL of AV binding buffer, and the analysis was run on a FACSCalibur (Becton Dickinson) flow cytometer. Through CellQuest software v.3.3 (Becton Dickinson), 50,000 events were acquired, and data were analyzed using Paint-a-Gate (Becton Dickinson). Results were expressed as a percentage of viable cells (AV−/PI−), early apoptotic (AV+/PI−), late apoptotic/necrotic (AV+/PI+), and necrotic cells (AV−/PI+) and represent the mean ± SEM of five independent experiments. 

For morphological analysis, 1 × 10^6^ cells from the different conditions were collected and seeded in glass slides. The smears were subjected to staining and analysis following the procedures defined by Lapa et al. [58].

### 4.12. Cell Cycle Analysis

Cell cycle analysis was performed using flow cytometry using a propidium iodide/RNase cell cycle analysis kit (Immunostep). Cells were grown in the absence or presence of 50 µM, 100 µM, 150 µM or 200 µM of AZD-7648 for 24 h or 48 h (KG-1). Then, 1 × 10^6^ cells were collected and washed with PBS by centrifugation for 5 min at 1000× *g*. The cells were fixed by incubation at 4 °C for 30 min with 200 μL of 70% ethanol solution added during vortex agitation. Afterwards, cells were washed with PBS, resuspended in 300 µL of propidium iodide/RNase, and analyzed on a FACSCalibur flow cytometer (Becton Dickinson). Cell cycle distribution was analyzed using ModFit^LT^ Software v. 2.0 (Verity Software House, Topsham, ME, USA). Results were expressed as the percentage of cells in the different cell cycle phases (G_0_/G_1_, S, and G_2_/M) according to propidium iodide fluorescence intensity and represent the mean ± SEM of five independent experiments. When present, apoptotic cells were identified as a sub-G_1_ population.

### 4.13. Apoptosis, DNA Damage, and Cell Proliferation Assessment

The Apoptosis, DNA damage, and Cell Proliferation Kit (BD Pharmingen, Franklin Lakes, NJ, USA) and anti-activated caspase 3 (BD Pharmingen) were used to analyze molecular markers of apoptosis, DNA damage, and cell proliferation, according to the manufacturer’s instructions. This method was exclusively performed in cell lines where the IC_50_ was reached, particularly LAMA-84, HEL, and KG-1. After 24 h or 48 h (KG-1) of treatment with different concentrations of AZD-7648, 1 × 10^6^ cells were washed, fixated, and permeabilized using BD Citofix Perm solution. Three hours before the end of this incubation, the cells were labeled with BrdU at 37 °C. Cells were stained with anti-cleaved PARP (PE), anti-activated caspase 3 (FITC), anti-H2AX (Alexa Fluor™ 647), and anti-BrdU (PerCP-Cy5.5). These markers were analyzed using flow cytometry in a FACSCalibur cytometer (Becton Dickinson). Results represent the mean ± SEM of five independent experiments and are expressed as the percentage of positive cells for each molecular marker.

### 4.14. Statistical Analysis 

Statistical analysis was performed in GraphPad Prism 9 software (version 9.0.0 for Windows; GraphPad Software, Inc. San Diego, CA, USA). The IC_50_ was calculated to the data concerning the exposure of cells at 24, 48, and 72 h, and the determination was performed by a non-linear curve fit dose-response. The normality of the samples was assessed using the Shapiro–Wilk test. Statistical significance was determined using a range of methods. These included unpaired *t*-tests (with or without Welch’s correction), one sample *t* test, Wilcoxon signed-rank test, the Mann–Whitney test, and ordinary one-way ANOVA. Following the ANOVA, either Tukey’s or Dunnett’s multiple comparisons tests were used. Moreover, Welch and Brown–Forsythe ANOVA, followed by Dunnett’s T3 multiple comparisons test, and Kruskal–Wallis test, followed by Dunn’s multiple comparisons test were also used. One-way ANOVA for repeated measures followed by Tukey’s multiple comparisons test or the Friedman test followed by Dunn’s multiple comparisons test was used to evaluate the effects of H_2_O_2_ through time. In this study, a significance level of *p* < 0.05 was considered statistically significant. All values were expressed as the mean ± SEM of the number of independent experiments (indicated in the figure legends).

## 5. Conclusions

In conclusion, these results using CML and AML models support the further investigation of the potential of AZD-7648 as a new therapy for myeloid leukemias. The tested cell lines presented different drug sensibilities, showing that the value of this therapy may depend, for example, on the patient’s genetic background, the expression of specific genes, and DDR efficiency and damage at the baseline level. Therefore, subsequent studies are necessary to determine with more precision what may confer sensitivity to this inhibitor. However, this work opens up the possibility of testing new combination schemes with other agents using lower doses that could synergistically induce a cytostatic and cytotoxic effect and overcome the resistance to AZD-7648. Lastly, studies that would assess the therapeutic potential of AZD-7648 in combination with conventional therapy may also help to elucidate this inhibitor’s promise further, considering that previous studies already showed the advantage of combining this drug with other DDR inhibitors, chemotherapy, or radiation [4,51,52,53,59,60].

## Figures and Tables

**Table 1:** Mutated DNA damage response genes, per pathway, in CML and AML cell lines.

DDR Pathway	CML		AML				
	K-562	LAMA-84	HEL	HL-60	KG-1	NB-4	THP-1
Base excision repair				*SMUG1*		*MBD4* *TDG*	
Other base excision repair and strand break joining factors	*APLF*	*PNKP*		*APLF*			
Nucleotide excision repair	*DDB1* *GTF2H1*		*ERCC5* *XAB2*		*RAD23A* *RPA3*		*GTF2H4*
Nucleotide-excision-repair-related	*ERCC6*				*UVSSA*		
Homologous recombination	*EME1*	*SPIDR*		*BARD1* *RAD54B*	*SMC6*	*BRAC1* *PAXIP1* *RAD51D* *RBBP8*	*RAD51D*
Non-homologous end joining	*PRKDC*		*PRKDC*	*PRKDC*		*XRCC5*	
Fanconi anemia		*FANCB* *FANCM*	*FANCD2*		*BRAC2* *FANCI*	*FANCD2*	*FANCD2* *FANCG* *PALB2*
Mismatch excision repair	*MSH4*				*MLH3* *MSH6*	*HFM1*	*MLH3* *MSH2* *MSH3* *PMS2*
Repair of DNA–protein crosslinks				*TDP1*			
DNA polymerases	*POLI*	*POLA1* *POLD1*	*POLE* *POLN*		*REV3L*	*POLD2* *POLQ* *REV1*	*POLD3* *POLM*
Chromatin structure and modification					*CHAF1A*		
Editing and processing nucleases	*DCLRE1A*		*ENDOV*		*DCLRE1A*		
Modulation of nucleotide pools			*NUDT1*				
Other conserved DNA damage response genes	*RAD17* *TP53*	*PER1* *RAD1* *TP53*	*TP53*	delTP53* PER1 *TP53BP1*	*CHEK1* *TOPBP1* *TP53*	*ATRIP* *CHEK1* *MDC1* *PER1* *TP53*	*TOPBP1* *TP53*
Ubiquitination and modification	*HERC2*			*RNF8*			*HERC2*
Genes defective in diseases associated with sensitivity to DNA damaging agents		*WRN*					*ATM* *TOP3A*
Other identified genes with known or suspected DNA repair function				*PRPF19*			*RECQL5*

The data on mutations in the DDR pathways were obtained from both the Catalogue of Somatic Mutations in Cancer (COSMIC) platform and the American Type Culture Collection (ATCC) (specifically for delTP53*); del, deleted.

**Table 2:** Baseline DNA and chromosomal damage and telomere length of normal human lymphocyte cell line (IMC), CML, and AML cell lines.

	CML			AML				
	IMC	K-562	LAMA-84	HEL	HL-60	KG-1	NB-4	THP-1
γH2AX expression (MIF)	19.0 ± 1.5	37.0 ± 1.7 **	29.3 ± 3.3	66.3 ± 5.9 ***	21.3 ± 2.2	25.3 ± 0.9	63.0 ± 1.5 ***	30.3 ± 0.9 *
BN cells with CDBs (%)	2.5 ± 0.2	7.2 ± 0.6 ***	8.8 ± 0.8 ***	3.6 ± 0.2	4.1 ± 0.3	5.4 ± 0.8 **	4.0 ± 0.3	5.9 ± 0.5 ***
Cells with MNi (%)	57.8 ± 6.8	19.8 ± 2.9 ***	24.5 ± 1.2 ***	25.5 ± 4.2 ***	30.9 ± 4.4 **	49.9 ± 5.0	30.4 ± 4.6 **	29.7 ± 7.1 **
Cells with NBUDs (%)	35.3 ± 6.3	52.2 ± 2.5	26.1 ± 2.3	52.8 ± 2.3	53.7 ± 4.0	27.5 ± 4.7	48.4 ± 4.0	57.9 ± 8.8 *
Cells with NPBs (%)	6.9 ± 3.7	28.0 ± 2.6 **	49.4 ± 2.3 ***	21.7 ± 4.3 *	15.4 ± 4.1	22.6 ± 1.7 *	21.2 ± 3.9 *	12.4 ± 4.5
Telomere length fold change (normalized to IMC cells)	1	0.16 ± 0.01 ***	0.25 ± 0.03 **	0.23 ± 0.04 **	0.16 ± 0.02 ***	0.09 ± 0.01 ***	0.26 ± 0.01 ***	0.38 ± 0.02 ***

γH2AX levels are expressed in mean fluorescence intensity (MFI). Chromosomal damage levels are expressed as the percentage (%) of binucleated (BN) cells presenting CDBs, and the frequency of different CDBs are expressed as the percentage (%) of BN cells displaying micronuclei (MNi), nuclear buds (NBUDs), or nucleoplasmic bridges (NPBs). For each experiment, 500 BN cells were scored. The telomere length of the cell lines was normalized to the telomere length of the IMC cell line. Results represent the mean ± SEM of 3 (γH2AX expression and telomere length) or 5 independent experiments. Data were statistically analyzed using one-way ANOVA followed by Dunnett’s multiple comparisons test or by one sample *t* test (telomere length) by comparison with IMC cell line. * *p* < 0.05, ** *p* < 0.01, and *** *p* < 0.001.

**Table 3:** Effect of AZD-7648 (AZD) in cell cycle distribution of CML and AML cell lines after 24 or 48 (KG-1) hours of treatment.

		Sub G_1_ (%)	G_0_/G_1_ (%)	S (%)	G_2_/M (%)
K-562	Control	1.6 ± 0.9	36.8 ± 2.4	48.6 ± 3.1	14.6 ± 1.3
	AZD 200 µM	4.2 ± 1.4	41.8 ± 3.5	49.4 ± 2.9	8.8 ± 1.7 *
LAMA-84	Control	1.8 ± 1.0	57.6 ± 1.6	30.6 ± 1.9	11.8 ± 0.8
	AZD 100 µM	4.6 ± 2.0	75.8 ± 1.5 ***	17.6 ± 1.6 ***	6.6 ± 0.8
	AZD 200 µM	5.4 ± 1.9	77.4 ± 1.3 ***	18.2 ± 1.8 ***	5.2 ± 0.8 **
HEL	Control	1.4 ± 0.4	41.0 ± 2.6	38.2 ± 2.5	20.8 ± 0.9
	AZD 50 µM	4.2 ± 0.9	71.4 ± 1.7 *	24.8 ± 1.9 *	3.8 ± 0.7
	AZD 150 µM	16.2 ± 2.8 **	72.0 ± 0.6 *	27.2 ± 0.6	0.8 ± 0.4 **
HL-60	Control	0.6 ± 0.4	38.6 ± 2.4	46.2 ± 1.7	15.2 ± 1.5
	AZD 200 µM	0.8 ± 0.2	83.2 ± 0.4 ***	11.4 ± 0.4 **	5.4 ± 0.4 **
KG-1	Control	0.6 ± 0.2	44.8 ± 0.7	41.8 ± 0.7	13.4 ± 0.2
	AZD 100 µM	1.2 ± 0.2	87.4 ± 0.4 ***	7.2 ± 0.6 ***	5.4 ± 0.2 ***
	AZD 200 µM	2.2 ± 0.2 **	82.4 ± 0.9	10.0 ± 0.8 ***	7.6 ± 0.2
NB-4	Control	0.2 ± 0.2	43.6 ± 1.6	48.4 ± 2.3	8.0 ± 1.9
	AZD 200 µM	1.4 ± 0.5	83.4 ± 0.7 ***	12.4 ± 0.6 ***	4.2 ± 0.6
THP-1	Control	0.2 ± 0.2	49.8 ± 1.4	38.2 ± 1.3	12.0 ± 0.7
	AZD 200 µM	1.6 ± 0.7	64.2 ± 0.7 ***	27.4 ± 0.4 **	8.4 ± 0.5 **

Data are expressed as a percentage (%) of cells per each cell cycle phase and represent mean ± SEM obtained from 5 independent experiments. Statistical analyses were performed by comparison with control, using one-way ANOVA and Dunnett’s multiple comparisons test, Kruskal–Wallis and Dunn’s multiple comparisons test, unpaired *t*-test, with or without Welch’s correction, and Mann–Whitney test. * *p* < 0.05, ** *p* < 0.01, and *** *p* < 0.001.

## Data Availability

All data generated or analyzed during this study are included in the published article.

## Supplementary Materials

The following supporting information can be downloaded at: https://www.mdpi.com/article/10.3390/ijms242015331/s1.
