# Supplementary material for: AZD-7648, a DNA-PK Inhibitor, Induces DNA Damage, Apoptosis, and Cell Cycle Arrest in Chronic and Acute Myeloid Leukemia Cells
Source: Int J Mol Sci. 2023 Oct 18;24(20):15331. doi: 10.3390/ijms242015331 (PMC10607085; doi:10.3390/ijms242015331)
Supplement: Supplementary file 1 [file ijms-24-15331-s001.zip › Supplementary Figure S1.pdf]

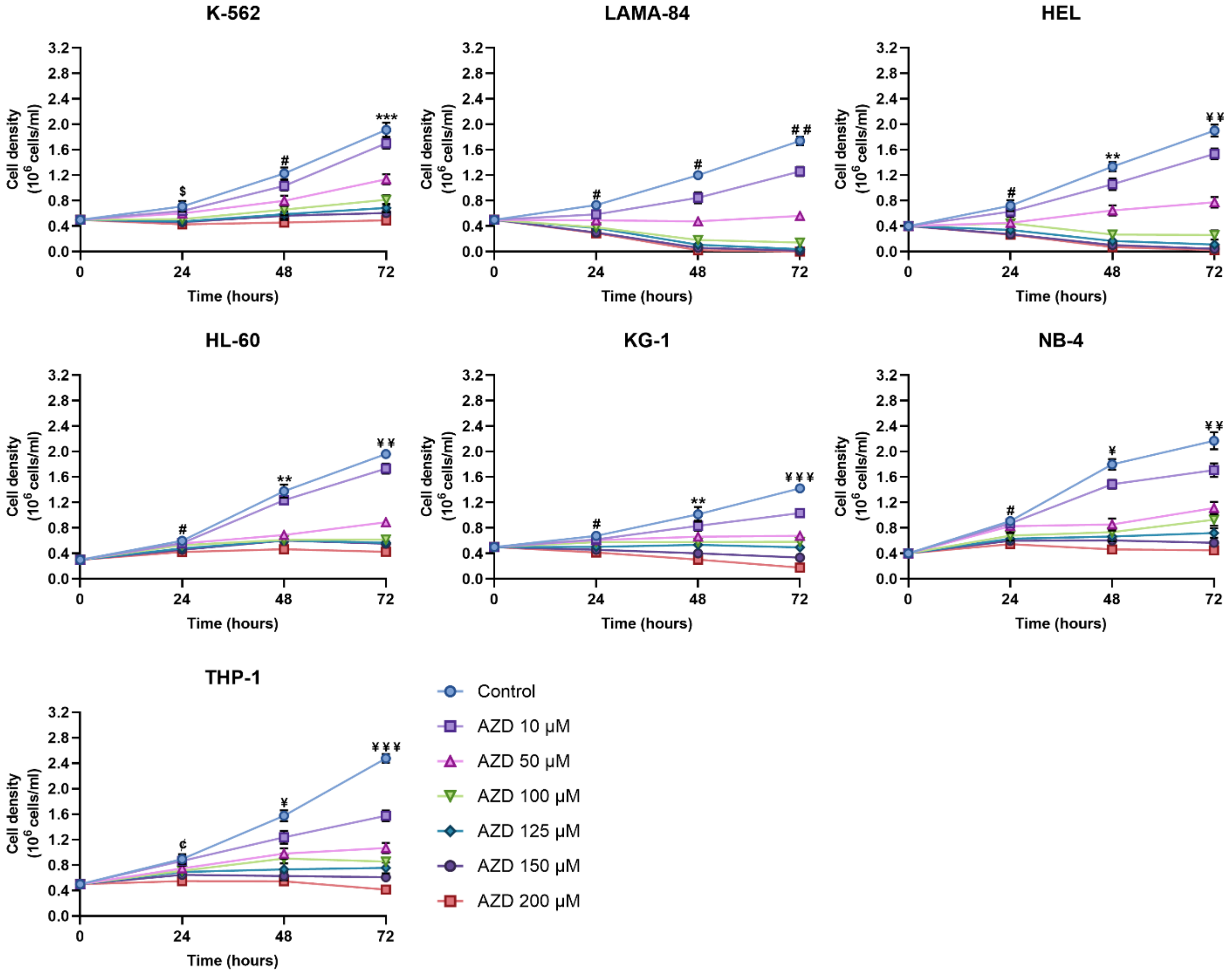

Supplementary Figure S1. The effect of AZD-7648 (AZD) on cell density in AML cell lines. Results are expressed as cellular density, represented as 10^6 cells/ml. Results represent the mean ± standard error of the mean (SEM) of 5 independent experiments. Data were statistically analyzed at each time point by comparison of the tested doses with control using ordinary one-way ANOVA followed by Dunnett's multiple comparisons test, Welch and Brown-Forsythe ANOVA followed by Dunnett's T3 multiple comparisons test, or Kruskal-Wallis test followed by Dunn's multiple comparisons test. ¥p<0.05, ¥¥*p*<0.01, and ¥¥¥*p*<0.001 (control vs 10, 50, 100, 125, 150, and 200 µM); ***p*<0.01, and ****p*<0.001 (control vs 50, 100, 125, 150, and 200 µM); $*p*<0.05 (control vs 100, 125, 150, and 200 µM); #*p*<0.05, and ##*p*<0.01 (control vs 125, 150 and 200 µM); ¢*p*<0.05 (control vs 150, and 200 µM).
